# What do Germans really think about health-nudges?

**DOI:** 10.1186/s12889-021-10808-7

**Published:** 2021-04-29

**Authors:** Mathias Krisam, Mona Maier, Rebecca Janßen, Johannes Krisam

**Affiliations:** 1 Charité University Clinic Berlin, Institute of Medical Sociology and Rehabilitation Science, Charitéplatz 1, 10117 Berlin, Germany; 2 Läuft GmbH, Behavioural Science, Wiesbadener Straße 42, 14197 Berlin, Germany; 3 ZEW Mannheim, P.O. Box 103443, D–68034 Mannheim, Germany; 4 Heidelberg University, Institute of Medical Biometry and Informatics, Heidelberg, Germany

## Abstract

**Background:**

In recent years, policymakers have increasingly used behaviourally informed policies, including ‘nudges’. They have been implemented to produce desirable social outcomes such as healthier eating and physical activity. In Germany, a small research team at the Federal Chancellery acts as the central unit to promote the introduction of nudges in the design of public life. Despite this, the nudging concept itself as well as the understanding around it has not spread widely among German citizens. When reporting about the concept, German media is often very critical of the concept.

**Methods:**

Using a for age, sex and educational level nationally representative online survey with 1000 participants, we investigate whether German citizens know about the concept of nudging. We also explore if they approve of the theoretical concept as well as a list of seven specific interventions regarding healthy eating and physical activity. A particular focus is placed on whether the level of approval is dependent on the target group of the intervention, as well as different intervention-initiators.

**Results:**

We find that nearly 80% of the respondents have never heard of nudging. However when being provided with a definition, we find that a strong majority (90%) supports the concept of nudging as well as all the specific interventions. Acceptance rates are higher if interventions are targeted at the general population compared to only children. All initiators – statutory health insurers, the government, private companies, and independent experts – are accepted as nudge initiators.

**Conclusion:**

Amongst Germans nudges are an accepted method to promote health behaviours. Policy makers from various fields in Germany should take that into account to improve future health policy.

## Background

In recent years, the application of the nudging concept has become more and more common in global politics and is used to shape interventions in the interests of citizens. Studies and (political) measures have been carried out especially in the area of nutrition, but also focussing on physical activity, alcohol and tobacco consumption as well as direct medical care [1, 2].

Despite the large growth in awareness and the numerous formations of Nudge-Units worldwide, knowledge and understanding of the Nudging concept has not spread widely among German citizens [3]. When Nudging is being discussed in the public domain, especially by the media, criticism is forthcoming. The media often portray Nudging as being manipulative and equate it with “psychological tricks”. They also make the claim that a state which uses Nudging treats its citizens like “sheep” and takes away their opportunity to learn from mistakes [4–8]. Furthermore, there is some criticism claiming that nudges are used to achieve goals that are in fact not useful or helpful to the person being nudged or to society [9].

However, despite the theoretical, conceptual, and scientific discussions around the pros and cons of Nudging, it should be a priority to ask the citizens themselves, rather than taking a stance over their heads, be it on either side.

When asking for citizens´ opinions on specific nudging examples (possibly without even mentioning the term ‘nudging’), a different picture emerges. In all studies that have been carried out so far, the majority of respondents support the introduction of those nudges that promote health [3, 10–19].

For a nudge to be perceived as a positive intervention it should be transparent and should not advise any illicit goals. For example, those nudges that target the unconscious (such as “subliminal advertising” against tobacco use in cinematographic films) are receiving very little acceptance [3, 10].

The originator of a nudge is of fundamental importance for its acceptance by citizens. In general, the acceptance of health nudges is higher or very high if they are put in place by independent experts such as physicians, psychologists or nutritionists, while the government as the initiator is regarded as rather critical [3]. Evers et al. [15] were able to show that the acceptability of nudges is highest in the case of independent experts, and lowest in the case of policy makers. Nudges initiated by the industry have greater approval rates than those introduced by policymakers.

So far, it has not been explored how statutory health insurers are perceived by the public as potential initiators of nudges. It is however known that statutory health insurers are trusted providers of health information, especially within the online channels [20, 21].

Besides the initiator of the nudge, the target group is also of importance for whether a nudge is accepted or not. Nudges that address children’s health have previously been shown to be more likely to be accepted than those that target the general public [3].

Based on these current findings, this study aims to investigate three different areas of research containing eight separate research questions with specific focus on Germany:

1. **To what extent do respondents know about the concept of Nudging?**
  **Q1**: Does only a minority of respondents know about the concept of nudging and can only a minority describe it in their own words?
2. **How high is the acceptance of health nudges in Germany?**
  **Q2**: Nudging is often criticised as a concept, but the specific interventions are not considered critical. Hence, is the approval for the specific interventions higher than the approval for the theoretical concept?
  **Q3**: If respondents are being framed with the conceptual description of nudging at the beginning of the survey, will their acceptance of the specific interventions listed afterwards be lower?
  **Q4**: A qualitative study which was conducted with only a small number of participants has shown that the acceptance of nudges is greater if their focus is the well-being of children [3]. We assume to replicate this finding with our quantitative approach.
  **Q5**: Are statutory health insurers more accepted as nudge initiators than the private industry and the state or government?
  **Q6**: Do independent experts receive the highest acceptance amongst all potential nudge-originators?
3. **How does the acceptance of nudges differ depending on different personal characteristics of the respondents?**
  **Q7**: Do people with children generally show higher acceptance than people without children [19]? Is this particularly evident for nudges aimed only at children?
  **Q8**: Are nudges generally more accepted if they are in line with the goals of the respective respondent [10]? Thus, can it be assumed that people with greater levels of health awareness have a higher acceptance of health-nudges?

## Methods

The survey was designed in a way to only include health nudges that either promote healthy eating or physical activity.

### Development and validation of the measurement tool

On our first version of the survey we got feedback from two field experts to gain face validity. After this design, the online-questionnaire was tested on 24 participants during the 3rd-9th of June 2019. During the pre-testing phase participants were asked for their feedback on the online-questionnaire. Based on this feedback changes were made to the questionnaire, mostly with regards to easier understandable wording of the questions including the descriptions of interventions. One question which asked for the participants’ preferred political party was omitted from the survey, as a few participants deemed it as too intrusive.

The survey questions were arranged into 5 sections: (1) quota-questions, (2) questions about the concept of nudging, (3) questions about 7 specific interventions in relation to two target groups, (4) questions about 4 interventions in relation to 4 nudge-initiators, (5) further socio-demographic questions.

In section 2, participants were asked if they had previously heard of “nudging”. Afterwards all respondents were shown a definition of nudging, and they were then asked to rate their level of approval of the concept of nudging based on that definition (see appendix 1). This entire section of the survey was only shown at the beginning of the survey for half of the respondents. The other half of the respondents answered this section towards the end of the survey, before the socio-demographic questions in section 5. Thereby we wanted to investigate whether respondents who were framed with the nudging concept before being asked about their level of acceptance for the specific interventions show different acceptance levels than those who were not framed.

In section 3, participants were asked about their level of acceptance of seven health nudges. All these nudges had the goal to either support healthier eating or to increase physical activity levels. The interventions were described in simple words without mentioning the word “nudge”. Each intervention was rated twice by the respondents: once for use on the general population, and once again for use exclusively on children. The order in which the nudges were presented to the participant was randomised to avoid any potential framing effects. The wording was as follows (originally in German): “In the following there will be seven different measures described to you, which all improve the health of adults or children. Please rate on a scale how much you agree or disagree with the introduction of these interventions.” Each of the following interventions was shown on a single screen. The description of the nudge was at the top, followed by the respondent being asked: “To what extent do you support this intervention if…”; the intervention was “aimed at everyone”; the intervention was only “aimed at children”. The interviewees then responded to both questions using a five-point Likert-scale ranging from “do not agree at all” to “fully agree”. The following nudge-interventions were part of the survey (see Appendix 2 for more detailed information on the questions):

1. Food traffic light labelling system
2. Warning labels on products with excessive salt or sugar content
3. Removal of unhealthy products at supermarket checkouts
4. Healthy products being positioned more easily accessible and visible in supermarkets
5. Restructuring of canteens
6. Non-commercial advertising of healthy products
7. Architecture of public spaces being designed to promote physical activity

In section 4, the acceptance of four different nudge-initiators was queried, respectively regarding four different interventions. The initiators were: government/ministries, statutory health insurers, private companies (e.g., food manufacturers, supermarkets, sports articles manufacturers), and independent experts (e.g., nutritionists, doctors). The wording was as follows: “In the previous section, several interventions were stated that improve the health of the population. Imagine that one actor was largely responsible for these interventions to be initiated. Four interventions were chosen as examples. Please rate on a scale how much you agree or disagree with the introduction of these interventions, depending on which initiator is responsible for the intervention.” Again, each of the following interventions was shown on a single screen. At the top was the description of the nudge and then the respondent was asked: “Please rate on a scale how much you agree or disagree with the introduction of these interventions, depending on which initiator is responsible for the intervention.” They were then presented with the same five-point Likert-scale as in the previous section. The following measures were evaluated for each initiator:

1. Healthy products being positioned more easily accessible and visible in supermarkets
2. Restructuring of canteens
3. Non-commercial advertising of healthy products
4. Architecture of public spaces being designed to promote physical activity

### Sampling procedure and sample size

Sampling and the survey were performed with the support of Qualtrics, which is a leading international ISO-certified market research company, in August and September 2019. To ensure the necessary level of rigor, we monitored and commented on each step of the sampling and survey implementation. To be able to compare our results with other studies we decided on a sample size of 1000 adults (≥ 18 years) living in Germany. Previous and referential studies have also worked with sample sizes of around 1000 participants per country [10, 22]. This sample was collected to be representative of the German population with respect to age, gender, and educational level (see Table 1). With this sample size at hand, it was possible to estimate the proportion of participants unfamiliar with the concept of nudging with a precision of 6.3% points in terms of the maximal width of the exact Clopper-Pearson 95% confidence interval.

**Table 1:** Sampling Quotas for Age, Gender, and Education

Variable	Category	Absolute and relative frequency *N* (%)	Reference values from German general population 2019^1 2^
Age	18–24	92 (9.2%)	9.2%
	25–34	153 (15.3%)	15.3%
	35–49	239 (23.9%)	23.9%
	50–64	265 (26.5%)	26.4%
	65+	251 (25.1%)	25.1%
Gender	Male	487 (48.7%)	47.9%
	Female	509 (50.9%)	50.1%
	Other	2 (0.2%)	1.0%
	Prefer not to answer	2 (0.2%)	1.0%
Education	ISCED 2	196 (19.6%)	19.6%
	ISCED 3–4	552 (55.2%)	55.2%
	ISCED 5–8	252 (25.2%)	25.2%

^1^: Age & Gender: EuroStat; https://ec.europa.eu/eurostat/en/web/products-datasets/-/DEMO_PJAN

^2^: Education: EuroStat; https://ec.europa.eu/eurostat/en/web/products-datasets/-/EDAT_LFS_9901

Qualtrics has a network of thousands of volunteers to take part in online surveys. Out of this database they contact the participants via email and announce the surveys. The surveys are then conducted in form of an online-questionnaire. Having all quota-relevant data about their volunteers, they can intentionally choose volunteers to fulfil the desired quota (age, gender, education in this study) and closed the survey for participants if certain quotas were already reached. In terms of region, quasi-representativeness was established with a maximum deviation of 2.8% per federal province.

For this study Qualtrics contacted 4266 participants. Out of these 1409 completed the survey successfully and 1000 respondents fulfilled the quality criteria after data cleansing.

#### Socio-demographics

Information was collected on socio-demographic variables and health awareness in sections 1 and 5. Those variables included gender (male/female/other/prefer not to say), age (in years), level of education, federal province, city size (number of inhabitants), and number of children. Health awareness was measured by a self-assessment health awareness item (“I take great care of my health”) presented as a 5-point Likert-scale ranging from “do not agree at all” to “fully agree”.

To ensure a high-quality sample, a range of validity and robustness checks were included when pooling the final data set. Apparently inattentive or careless respondents were excluded by employing a time filter (sorting out respondents who used less than half of the average time needed to answer the survey). Respondents were forced to answer all questions (i.e., no skipping and “cherry picking”), and only fully completed questionnaires were accepted. Respondents who gave the same answers to all questions in section 3 and/or section 4 were identified as “straight liners” and were replaced with new respondents.

Field work started with a soft launch of 10% of the respondents on the 12th of July 2019. Results were checked for consistency, validity, and robustness. Minor adaptations were made for the remaining 90% of the sampling which started on the 23rd of July 2019. Field time ended on the 24th of September 2019 (hence, overall field time was about nine weeks).

### Analysis

**Fig. 1:**
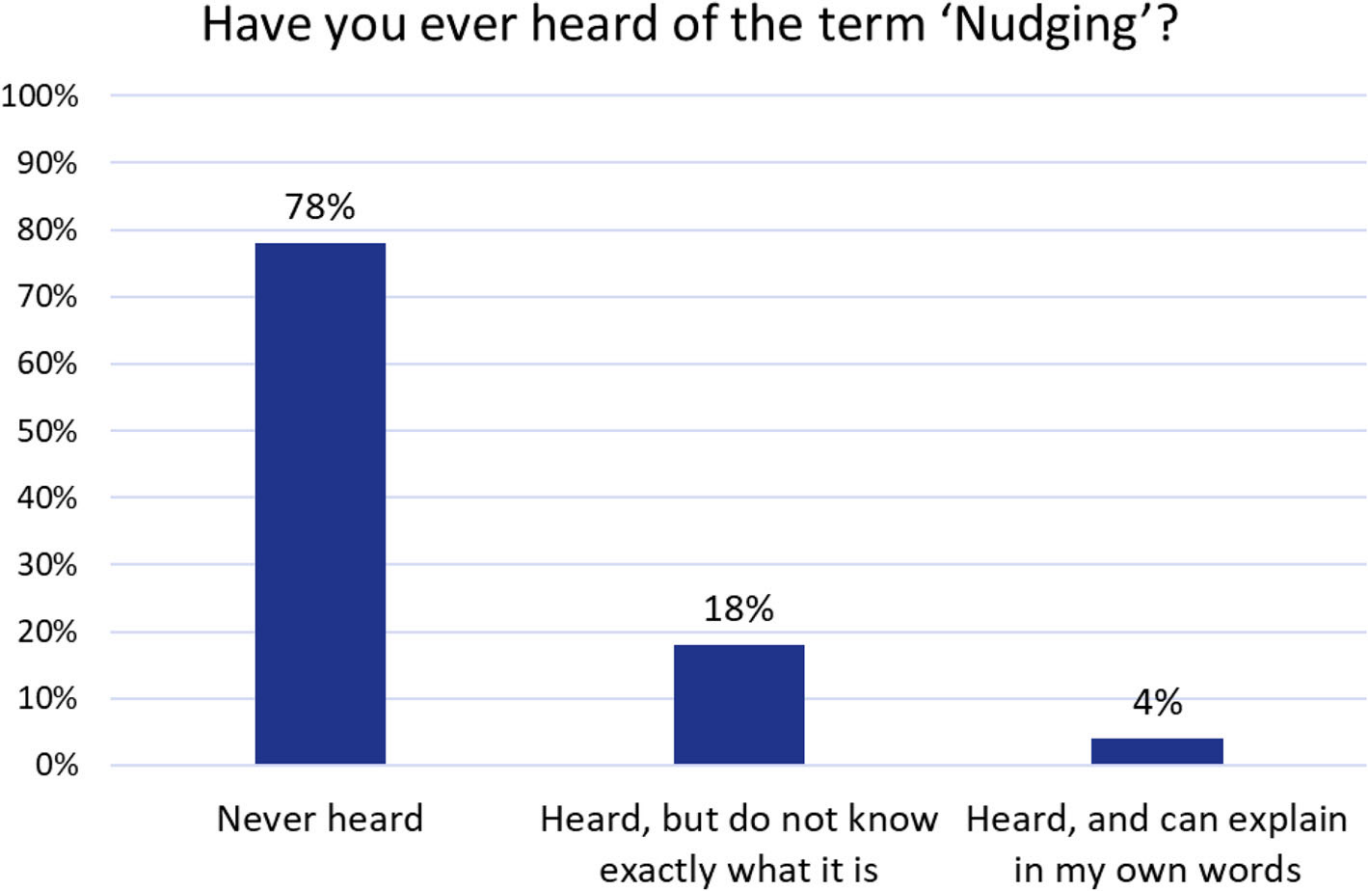
Awareness of the nudging concept

The analysis of the data was done both descriptively and by means of correlation analyses and (descriptive) statistical tests. In order to investigate the *first area of research* about the extent to which respondents know about the concept of nudging the proportions of the different answer categories of two questions were established and presented (Fig. 1). The questions which were asked to assess the first research question were (1) whether the participant had heard of Nudging and (2) whether they could describe the term in their own words.

To investigate the *second area of research* about the level of acceptance towards health nudges in Germany, several steps and several methodological approaches were taken, which are explained in the following. Since we measured the participants’ acceptance of both the theoretical concept and the specific interventions, we obtained dependent samples of variables with a 5-point ordinal scale for each participant. Thus, Wilcoxon signed rank tests were used to calculate (descriptive) *p*-values in order to examine whether the evaluations differed for the specific interventions compared to the theoretical concept (research question 2). *P*-values were also calculated to see whether statutory health insurers are more accepted and therefore receive higher ratings as nudge originators compared to the private industry and the state or the government (research question 3). In this instance, Friedman tests were conducted since more than two dependent variables with ordinal scale were compared. Similarly, *p*-values were calculated using Friedman tests to compare the group of independent experts as nudge initiators to government/ministries, statutory health insurers, and private companies (research question 4). Wilcoxon signed rank tests were used to investigate whether the evaluations for interventions differed if the target group was only children or the entire population (research question 5). To assess whether those respondents who were framed with the concept of nudging at the beginning of the survey rated the interventions differently compared to those respondents who were not framed (research question 6), we determined *p*-values of the Mann-Whitney-U-test, since we compared the two independent samples of framed and non-framed participants.

To investigate the *third area of research* about how the level of acceptance is dependent on personal characteristics, *p*-values based on the Mann-Whitney-U-test for two independent samples were calculated. Those enabled us to explore whether people with children show a higher acceptance of the theoretical concept as well as the different interventions compared to people who do not have children (hypothesis 7). Then, Spearman correlations were calculated to compare how different levels of health awareness are associated with approval rates of the concept as well as interventions (hypothesis 8). Finally, an exploratory analysis was conducted in which any potential effects of gender, age, education, living in urban vs rural areas as well as in east- vs west-Germany on the rating of the concept as well as the interventions were investigated. For gender, urban vs rural, and east- vs west *p*-values based on the Mann-Whitney-U-Test were calculated, since we compared two independent samples for each comparison, respectively. To investigate age group as well as level of education, Spearman correlations were calculated. Cronbach’s alpha was calculated for the items of our survey in order to assess internal consistency. Due to the exploratory character of the trial, all resulting p-values are only of descriptive nature and have no confirmatory value. Thus, no adjustment for multiple testing was performed. *P*-values smaller than 0.05 were regarded as statistically significant.

All analyses were carried out using the statistical software R v 3.6 (ww.r-project.org).

## Results

### Level of awareness and appraisal of the nudging concept as well as nudging interventions in general

Nudging is largely unknown among German citizens. 77.6% (95%-CI = [74.89%; 80.15%)] of respondents have never heard of nudging, and only 4.1% (95%-CI = [2.96%; 5.52%]) say they can explain nudging in their own words (Fig. 1). This confirms that only a minority of respondents knows the concept of nudging and can describe it in their own words.

**Fig. 2:**
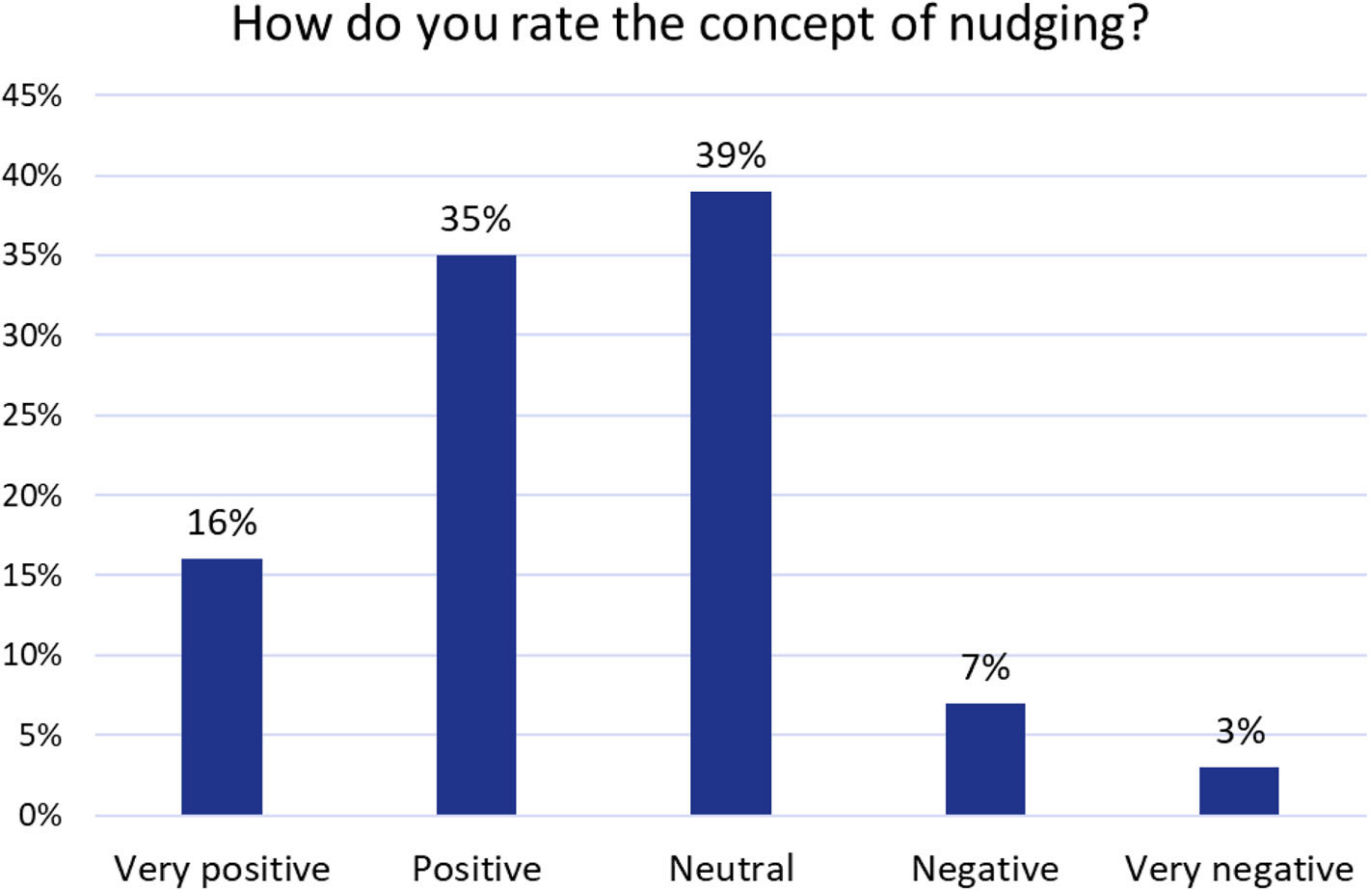
Evaluation of the nudging concept

Contrary to the mostly negative media coverage, most citizens approve of the nudging concept (after being provided with a definition). The vast majority (90%) of all respondents rate the nudging concept as (very) positive (51%) or neutral (39%) and only a minority (3%) consider it as very negative (Fig. 2).

**Fig. 3:**
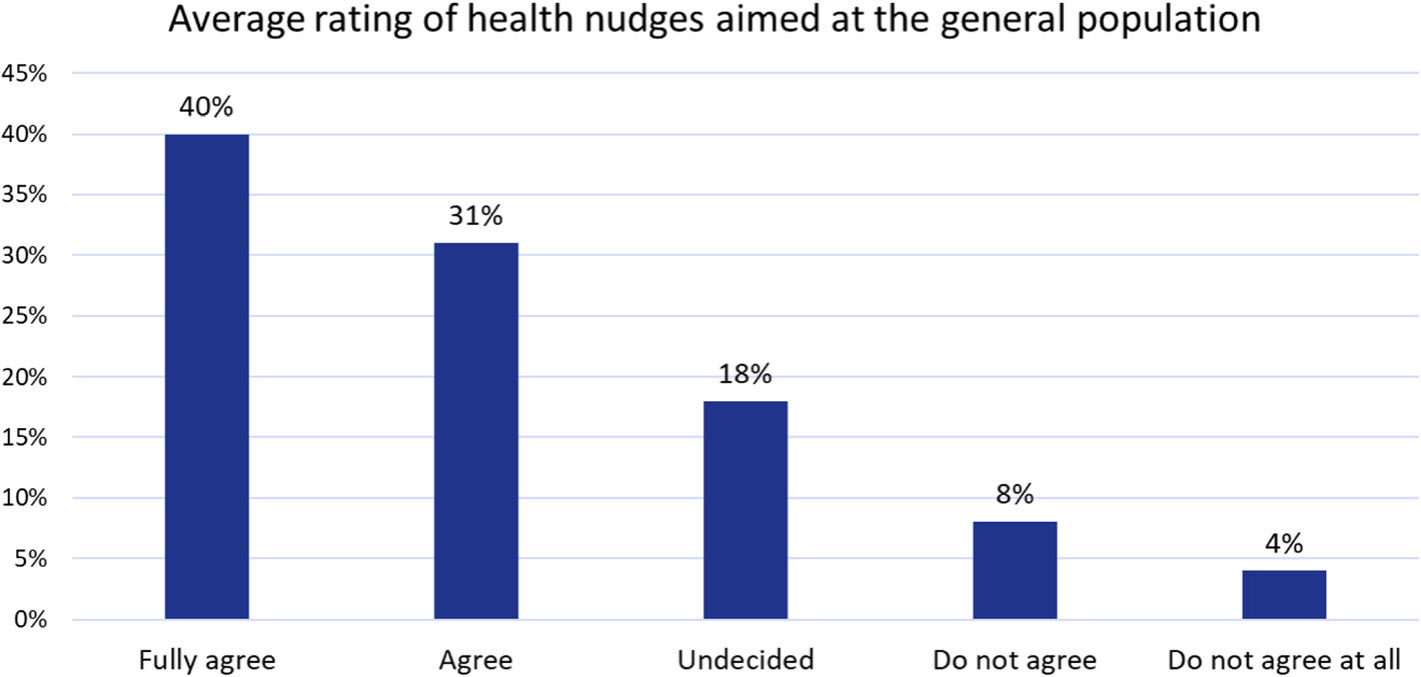
Average evaluation of specific nudging interventions

When rating specific interventions rather than the concept of nudging, on average, 89% of respondents evaluate interventions targeted at the general population as positive (71%) or neutral (18%). Together with the already high acceptance of the nudging concept itself (Fig. 2), it is evident that the German population is open for health nudges (Fig. 3).

**Fig. 4:**
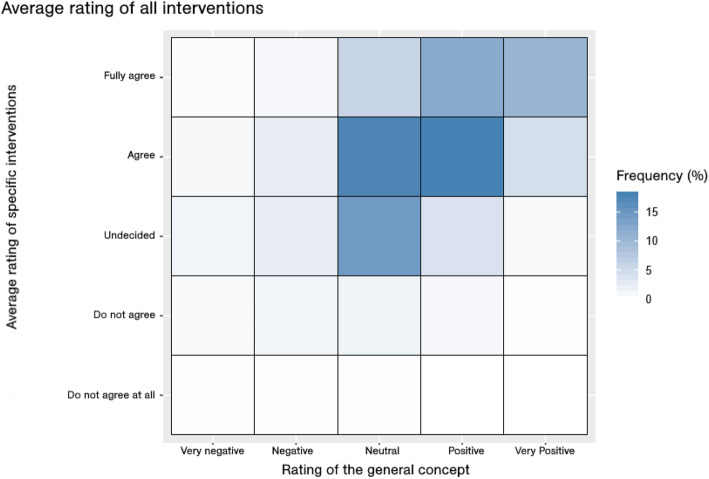
Agreement between the ratings of the average of the specific interventions and of the theoretical concept

Figure 4 shows in colour coding the level of conformity between the ratings of the specific interventions (averaged across the seven interventions) and the theoretical concept. For an equal rating of both, the blue fields would have to be mostly on the diagonal. If the specific interventions received better ratings than the theoretical concept, the blue fields are above the diagonal, otherwise below. The plot shows that the specific interventions were rated better than the general concept. The *p*-value for a difference in the mean score of the specific interventions and the theoretical concept is *p* < 0.0001. Therefore, the **second research question** (specific nudging interventions have a higher acceptance score than the general concept) could be positively answered.

**The third research question**, which stated that if respondents are framed by the conceptual description of nudging at the beginning of the survey, their acceptance of the specific interventions listed afterwards will be lower, had to be negatively answered. The *p*-values of the Mann-Whitney U test in Table 2 do not indicate that being framed with the concept of nudging at the beginning of the survey influences the acceptance ratings of the interventions. Significant differences were found only in the intervention “traffic light labelling system” (children) and “supermarket general” (children). Interestingly, in the case of the framed participants, the theoretical concept was rated significantly worse than by those who rated the concept at the end of the survey.

**Table 2:** Comparison of ratings of interventions between framed and non-framed participants / participants with and without children

Intervention	Difference Framing - no Framing		Difference participants with – and without children	
	Test statistics W^1^	*p*-value^1^ and direction of effect^2^	Test statistics W^1^	*p*-value^1^ and direction of effect^2^
Theoretical concept	106,294.5	*p* < 0.0001 (NF)	112,077.5	*p* = 0.0061 (C)
Traffic light labelling system	119,238.0	*p* = 0.1786 (NF)	119,146.0	*p* = 0.2702 (C)
Traffic light labelling system (children)	137,450.0	*p* = 0.0053 (F)	120,777.0	*p* = 0.4900 (C)
Warning labels	124,520.0	*p* = 0.9108 (NF)	120,511.5	*p* = 0.4334 (C)
Warning labels (children)	126,136.0	*p* = 0.7990 (F)	125,639.0	*p* = 0.6867 (NC)
Supermarket checkouts	129,481.0	*p* = 0.3095 (F)	111,942.0	*p* = 0.0067 (C)
Supermarket checkouts (children)	129,869.5	*p* = 0.2750 (F)	111,586.5	*p* = 0.0058 (C)
Supermarket general	129,305.0	*p* = 0.3193 (F)	114,684.5	*p* = 0.0332 (C)
Supermarket general (children)	135,597.5	*p* = 0.0174 (F)	116,119.0	*p* = 0.0815 (C)
Canteens	125,357.5	*p* = 0.9345 (F)	118,090.0	*p* = 0.1830 (C)
Canteens (children)	132,674.5	*p* = 0.0851 (F)	116,786.5	*p* = 0.1115 (C)
Advertisement	129,521.0	*p* = 0.3021 (F)	116,213.0	*p* = 0.0799 (C)
Advertisement (children)	130,463.5	*p* = 0.2196 (F)	126,463.5	*p* = 0.5550 (NC)
Environment	126,346.0	*p* = 0.7528 (F)	119,343.5	*p* = 0.2895 (C)
Environment (children)	123,637.5	*p* = 0.7531 (NF)	115,604.0	*p* = 0.0557 (C)

*F* Better rating if framed, *NF* Better rating if not framed, *C* better rating if respondent has children, *NC* better rating if respondent has no children

^1^: Provided test statistics and *p*-values are based on two-sided Mann-Whitney U tests

^2^: The direction of the effect was assessed by conducting 2 1-sided Mann-Whitney U tests, each pointing in a different direction. The direction with the smaller *p*-value was then considered as the direction of the observed effect

### Level of acceptance for specific nudging interventions depending on target group and nudge-initiator

Nudging interventions targeting only children were rated significantly more negatively than nudges addressing the general population. This difference in ratings between both target groups is statistically significant for all interventions (*p* < 0.001). **Research question 4** (acceptance of nudges is greater if their focus is on the well-being of children) was therefore negatively answered.

**Fig. 5:**
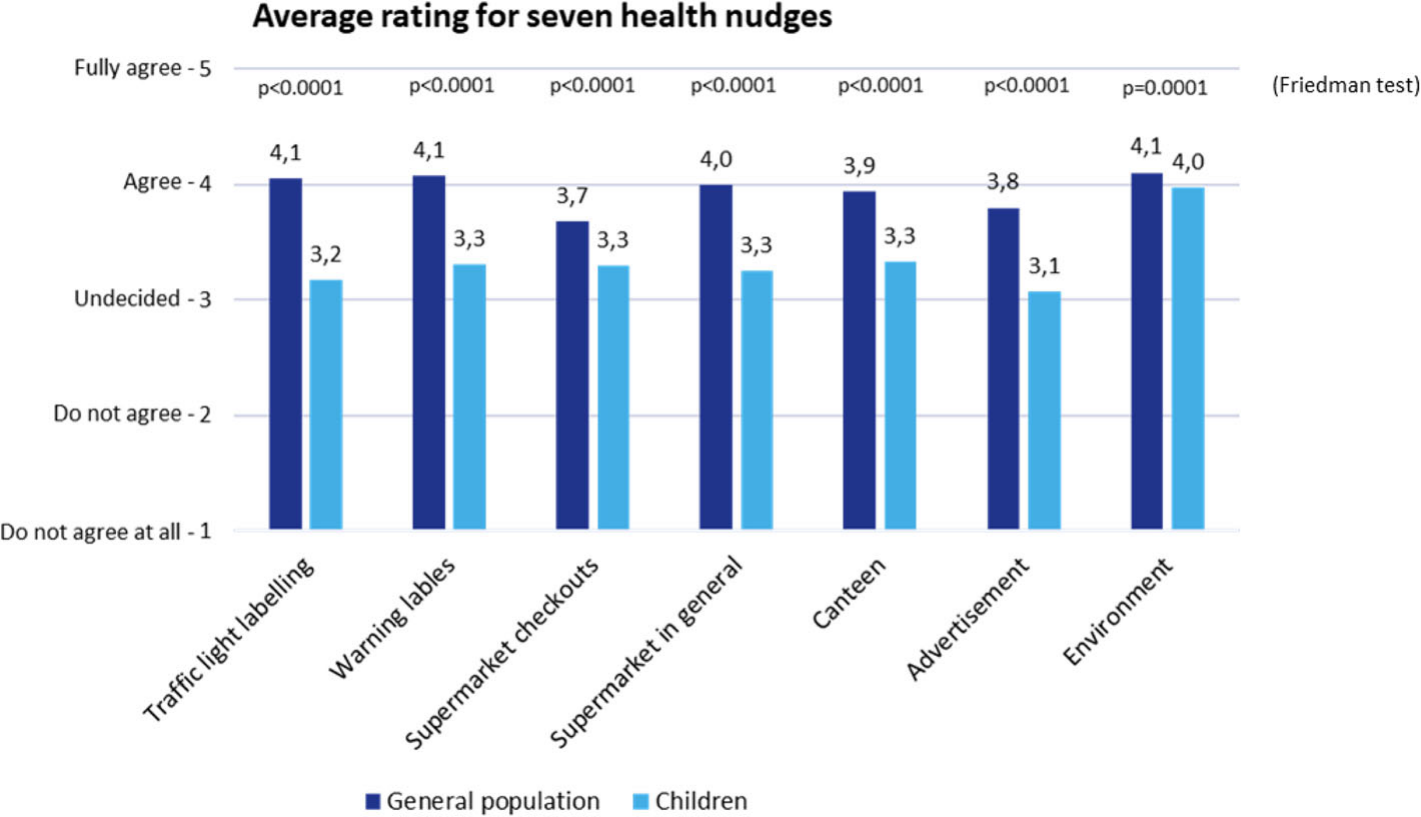
Average rating of health nudges

For different nudge initiators, the average approval rating for different health nudges is positive (“agree”) (Fig. 5). This means that the government/ministries, statutory health insurances, private companies and independent experts are all accepted as originators of health nudges.

**Fig. 6:**
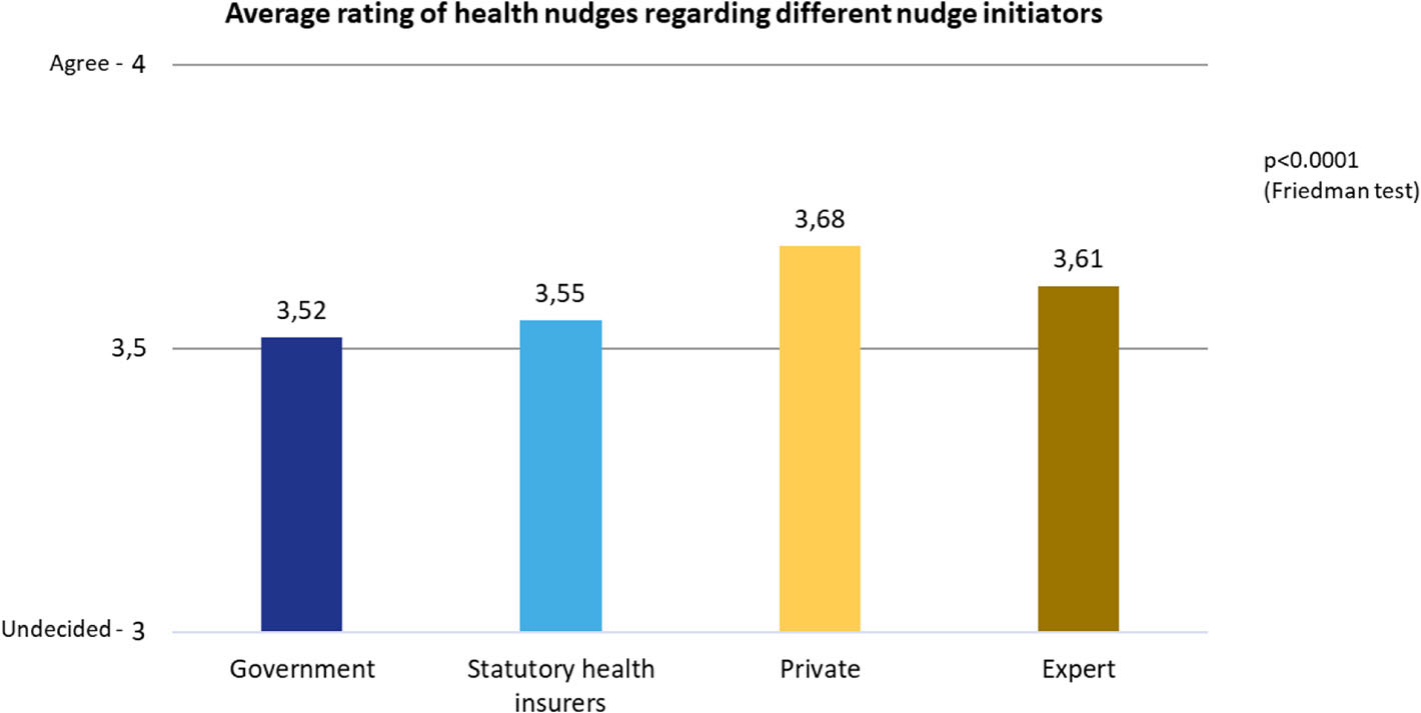
Average rating of health nudges regarding different nudge initiators

**Fig. 7:**
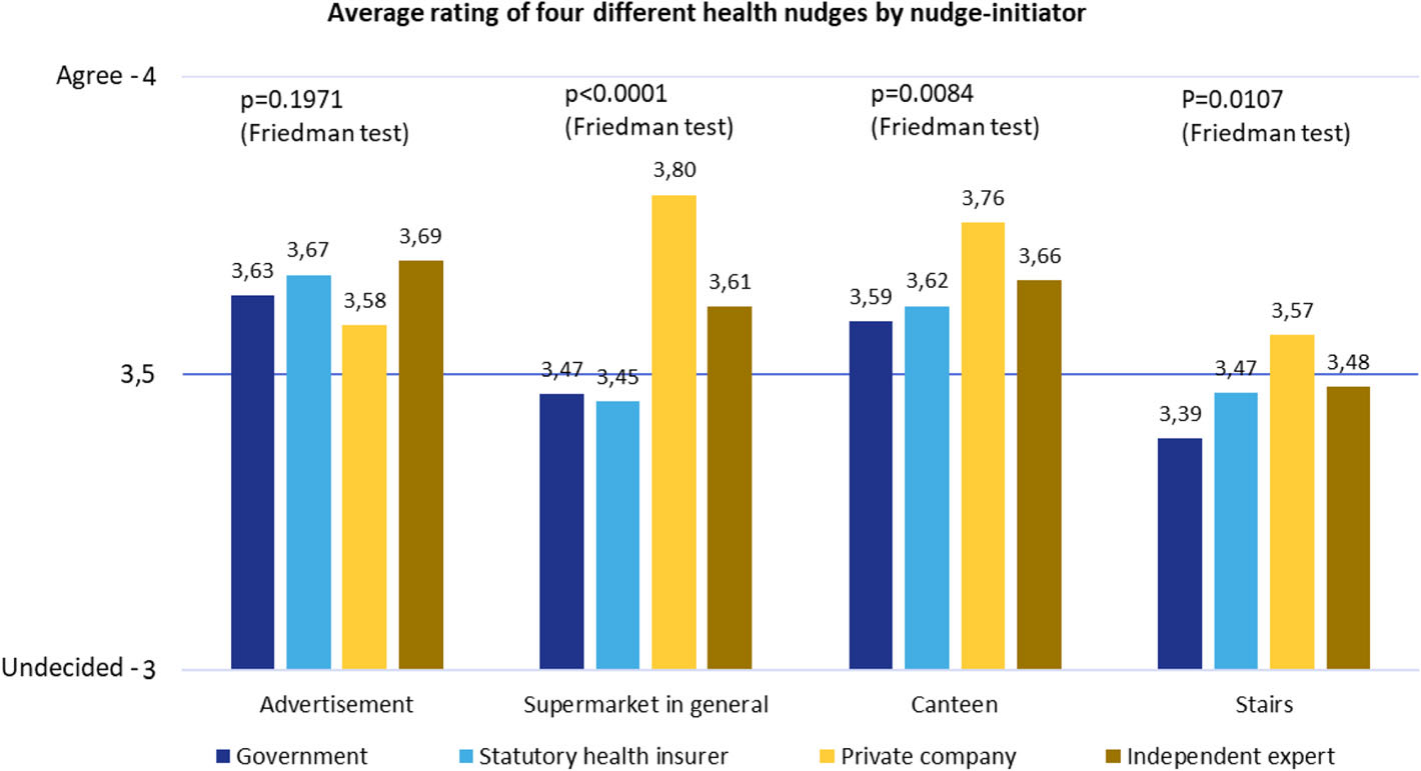
Average rating of specific health nudges regarding different nudge initiators

Compared to the government, the statutory health insurers score significantly better only in the case of the “stairs” intervention (Fig. 6 and Fig. 7). Compared to private companies, the health insurances perform better only in the “advertising” intervention, but in the case of the other three interventions private companies were rated significantly better than the statutory health insurers. **Research question 5** (statutory health insurers are more accepted as nudge initiators than the private industry and the state or government) can thus only be partly answered positively.

Experts were rated significantly better than the government in the “supermarket” and “stairs” interventions. In the “supermarket” intervention, they were rated significantly better than the health insurances. In the case of the “advertising” intervention, they were rated significantly better than private companies, but significantly worse compared to private companies in all three other measures. **Research question 6** (independent experts receive highest acceptance) could therefore also partly be answered positively.

### Level of acceptance for the concept of nudging and specific nudging interventions depending on personal characteristics

#### Children vs no children

Only the theoretical concept of nudging and both interventions in the supermarket (“Checkout” and “General”) were rated significantly better by people with children (see Table 2). Therefore, **research question 7** (people with children show higher acceptance rates than people without children) cannot be answered fully positively.

#### Health awareness

**Research question 8**, investigating whether people with a greater level of health awareness have a higher acceptance of health-nudges could not be confirmed. The spearman correlation between the rating of the concept of nudging and the health awareness question is with *ρ* = 0.2116 (95% CI = [0.1509; 0.2706]), rather low. The spearman correlation between the average rating of all interventions and the health awareness question is with *ρ* = 0.3231 (95%-CI = [0.2650; 0.3789]) also rather low.

#### Other sociodemographic factors: gender, age, education, living in urban vs rural areas

We also looked at whether other sociodemographic factors had an association with the acceptance of nudging. Our data shows that women, older people and people living in rural areas rate the concept of nudging significantly higher than men, younger people and people living in urban areas, respectively. People holding an educational qualification rate the concept of nudging higher than those who do not hold an educational qualification. This difference was however not statistically significant and holding an educational qualification did not show a systematic effect on the rating of the interventions.

#### Internal consistency of the survey

Internal consistency amounted to α = 0.91 (95%-CI = [0.9, 0.92]) which can be deemed as satisfactorily high [23].

## Discussion

Even though the majority of Germans does not know of nudging there is strong support for the concept itself as well as for specific interventions based on the concept. This is even the case despite the predominantly negative media coverage. These findings are in line with previous evidence that health nudges typically enjoy majority approval [3, 10, 12, 19].

The approval for specific interventions is higher than the approval for the theoretical concept of nudging. Although we must consider that all participants – despite the definition and the given examples – might not reach exactly the same understanding. When it comes to implementing nudging interventions, it might be more appropriate to avoid using the word “nudging” in communication for the time being. It should be considered whether a more positive communication strategy around the term “nudging” could be sought out. This could include finding a new unbiased (German) term. Respondents who rated the theoretical concept after rating specific interventions showed significantly higher approval rates for the concept compared to those who rated the concept beforehand. This can be indicative that providing people with a number of different specific nudge examples might make them more appreciative of the concept itself.

In contrast to previous research by Junghans et al. [3] we found that nudges which target the general population are more accepted than nudges that only target children. From our findings we suggest that health-nudges should be introduced to the whole population rather than to certain groups.

All four different nudge-initiators that were considered within the survey received high acceptance rates. Statutory health insurances and the government or ministries respectively should take the concept of nudging into consideration when designing interventions. It was particularly surprising that private companies receive such positive approval ratings as nudge-initiators. There are many opportunities for the private sector to use health-nudges for their own employees and customers. Apart from independent projects, this could also result in interesting constellations for public-private-partnerships, which could, for example, be realised as part of corporate social responsibility projects. The underlying reasons for this surprisingly positive rating of private companies in the role of nudge initiators could not be detected in the context of this study. Therefore further qualitative research should be conducted.

When looking at the initiators and interventions in more detail, supermarkets could initiate health promoting measures in their stores, especially if they want to target customers with children. The same applies to the design of canteens which could be changed by their operators or the respective company where the canteen is located. Healthier options could be presented more attractively (e.g., colours, nice dishes) and easier to reach (e.g., on eye level, closer to the customer, at the checkout). In terms of motivational posters for increasing the use of stairs in public spaces, the highest level of acceptance is granted to private companies (e.g., sporting goods manufacturers). Measures could involve nudges to use the stairs instead of the escalator or lift, prompts to walk to the next bus stop or reminders to change from sedentary to standing or walking activities. Despite no significant differences in approval rates for non-commercial advertising of healthy products, this is recommended to be initiated by public authorities (government, statutory health insurers) or should at least be accompanied by independent experts to uphold credibility. For the public sector and the statutory health insurance companies, the joint external communication via “independent experts” is recommended, as this can increase the acceptance among citizens.

Given the results that nudges receive a higher acceptance rates from women, older people and people living in rural areas, nudging interventions could be introduced to initially target these groups to have a high acceptance rate from the beginning in the German population.

With previous findings and the study presented here, we are only at the beginning of the urgently needed discussion around behavioural interventions in Germany. Many questions around the topic have been answered or have at least been discussed within this research. However, one thing is clear: The German population appears to be in favour of the implementation of health-nudges.

## Limitations

Given the fact that so far no tool existed to measure nudging acceptance, we need to take into account that this survey has been developed by ourselves we cannot guarantee construct validity of the survey. Furthermore, we did not test if the participants understood the concept of nudging correctly and did answer accordingly.

## Conclusion

Although only very few Germans are familiar with the term ‘Nudging’, the vast majority supports the application of health promoting nudges. Differences arise according to the specific measure and initiator. Nudges targeting the general population are more accepted than nudges that only target children.

## Appendix

### Appendix 1: Definition of Nudging

A nudge is a behavioural economic method that seeks to influence people’s behaviour in a predictable way. It does not rely on prohibitions and mandates and economic incentives remain unchanged, instead it changes the “choice architecture”. This refers to the physical, social and psychological environment that influences a decision. The goal of nudging should always be to increase welfare. An example of a health nudge would be to bring healthy foods closer to eye level or to replace unhealthy products (such as chocolate) at supermarket checkouts with healthy products (such as fruit), so that more people choose fruit rather than chocolate.

### Appendix 2: Detailed information on survey questions

**Table:**

1. A food traffic light labelling system is introduced that promotes a healthy diet (red = unhealthy food, yellow = neither really healthy nor really unhealthy food, green = healthy food). a. This food traffic light is applied to all foods b. This food traffic light is only used on foods that are aimed at children (for example, sweets, baby foods, children’s cereals, “Quetschies”)	
2. Warning labels are introduced on products with excessive salt or sugar content (e.g. ready meals such as frozen pizza or potato chips). a. These warnings are displayed on all products with excessive salt or sugar content b. These warnings are displayed only on products with excessive salt or sugar content that are aimed at children	
3. At supermarket checkouts, unhealthy products (such as alcohol, cigarettes, and sweets) are removed, but can still be bought elsewhere in the supermarket. In order to help customers buy healthy products, such products such as fruit or mineral water can now be found at the checkouts. a. This affects all unhealthy products b. This only applies to products that are aimed at children	
4. Healthy products are more easily and visibly positioned in supermarkets generally (e.g. at eye level, closer to the consumer, in prominent places, or with special indications), so that consumers increasingly turn to healthy foods. a. This affects all unhealthy products b. This only applies to products that are aimed at children	
5. To promote a healthy diet, canteens are restructured so that healthy food and its payment are made more accessible (e.g. closer to the consumer or directly in front of the cashier) and visible (e.g. mineral water at eye level instead of soft drinks (e.g. coca) Cola)). a. This is implemented in all canteens (public canteens, company and school canteens) b. This is implemented exclusively in school canteens.	
6. To promote a healthy diet, non-commercial advertising on television, in the cinema and on the Internet will be introduced, which will show the consumption of healthy foods such as fruits and vegetables. a. This non-commercial advertising addresses all users b. This non-commercial advertising is aimed exclusively at children	
7. From now on, our environment will be improved to promote more exercise, such as safe pedestrian and cycle paths, as well as easy access to green and recreational areas or exercise opportunities. a. This is implemented in cities and municipalities b. This is implemented in schools (for example by prominently placed stairs, the arrangement of important rooms on different floors or the introduction of “movement breaks”	
